# Peroral endoscopic myotomy for sigmoid-type achalasia after Heller myotomy and endoscopic submucosal dissection for an early esophageal cancer in a single endoscopic procedure

**DOI:** 10.1055/a-2418-0711

**Published:** 2024-10-02

**Authors:** Can Zhao, Longsong Li, Ningli Chai

**Affiliations:** 1 651943 Gastroenterology, Chinese PLA General Hospital First Medical Center, Beijing, China

**Fig. 1:**
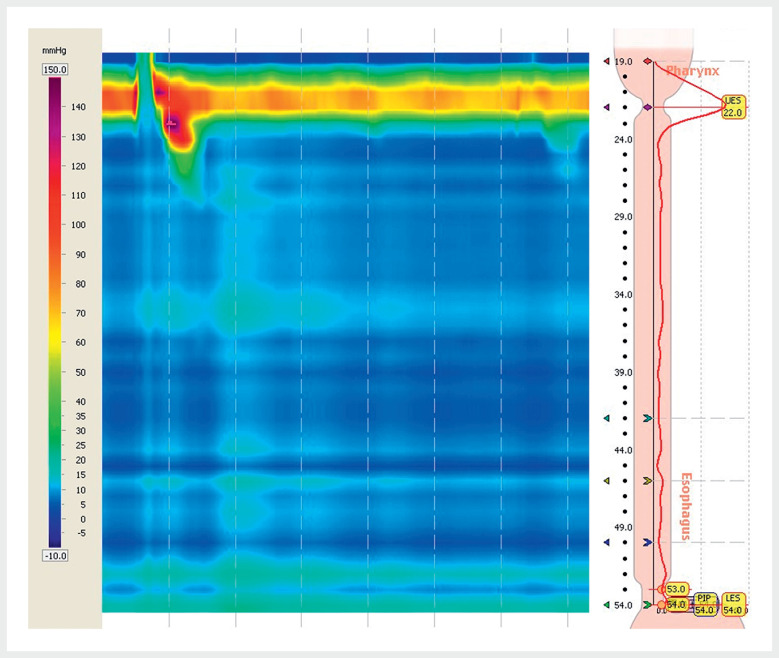
High resolution esophageal manometry confirming the diagnosis of achalasia (Chicago classification type I).

**Fig. 2:**
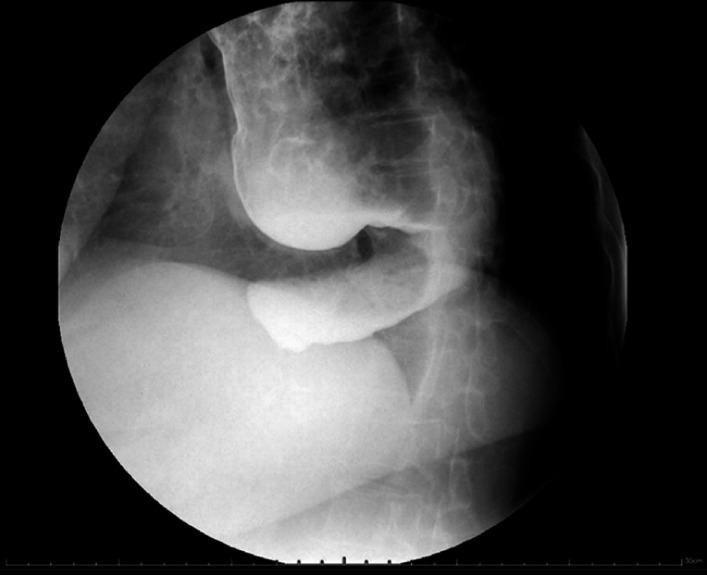
Radiographic image showing the sigmoid esophagus.

A 51-year-old man with achalasia, whose symptoms had not improved significantly after Heller myotomy performed in another hospital, was definitively diagnosed with achalasia in our hospital using high resolution esophageal manometry ( Fig. 1 ); a sigmoid esophagus was visible on esophagography ( Fig. 2 ), and the patientʼs esophageal lumen was seen to be extremely dilated and significantly twisted on endoscopic examination, which made it difficult to pass the endoscope through the cardia.

**Fig. 3:**
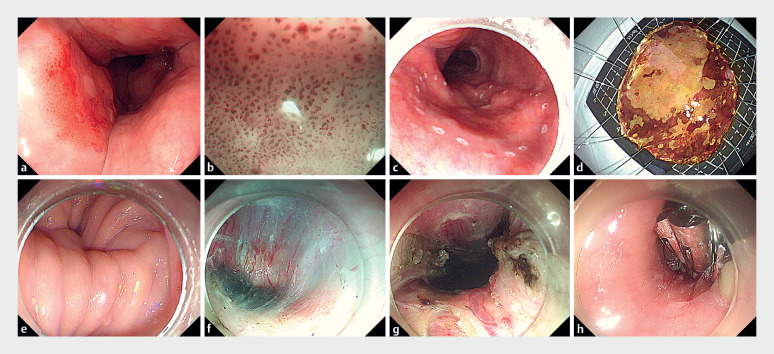
Endoscopic images of the combined endoscopic submucosal dissection and peroral endoscopic myotomy procedures showing: **a** the area that was suspicious for early cancer; **b** brownish discoloration of the lesion on narrow-band imaging; **c** marks placed around the lesion prior to dissection; **d** the macroscopic appearance of the resected lesion; **e** the severely twisted esophageal cavity; **f, g** construction of the submucosal tunnel and subsequent myotomy; **h** endoscopic clips placed to close the tunnel.

Unexpectedly, a rough mucosa was found 30 cm from the incisors. Narrow-band imaging (NBI) showed a brownish discoloration, suggesting early esophageal cancer. The patient requested simultaneous treatment of the esophageal lesion. We first performed endoscopic submucosal dissection (ESD) to resect the lesion, then used the short-tunnel technique to perform peroral endoscopic myotomy (POEM) ( Fig. 3 ). Intraoperative hemostasis was strictly observed. The entire procedure took 53 minutes and the patientʼs symptoms were significantly improved postoperatively ( Video 1 ).

Endoscopic submucosal dissection for an early esophageal cancer and peroral endoscopic myotomy for achalasia are performed during a single endoscopic procedure.Video 1

The timing of the achalasia procedure proved very timely for this patient, resolving the symptoms of his dysphagia and also uncovering the previously unknown early esophageal cancer, which was treated within the same session, thereby reducing the number of sessions of general anesthesia required and lowering his surgical risks. The short-tunnel technique for POEM was suitable in this case and did not affect the newly created ESD wound, with all of the treatments achieving positive effects.

The combined treatments of ESD for early esophageal cancer and POEM for severe achalasia of the cardia proved to be feasible and effective in this patient.

Endoscopy_UCTN_Code_TTT_1AO_2AN

